# Effect of early interventions with manual lymphatic drainage and rehabilitation exercise on morbidity and lymphedema in patients with oral cavity cancer

**DOI:** 10.1097/MD.0000000000030910

**Published:** 2022-10-21

**Authors:** Kuo-Yang Tsai, Su-Fen Liao, Kuan-Lin Chen, Hao-Wei Tang, Hsin-Ya Huang

**Affiliations:** a Department of Oral and Maxillofacial Surgery, Show Chwan Memorial Hospital, Changhua, Taiwan; b Department of Physical Medicine and Rehabilitation, Changhua Christian Hospital, Changhua, Taiwan; c Division of Pediatric Rehabilitation, Changhua Christian Children’s Hospital, Changhua, Taiwan; d Department of Post-Baccalaureate Medicine, College of Medicine, National Chung Hsing University, Taichung, Taiwan; e Department of Physical Medicine and Rehabilitation, Yuan Rung Medical Corporation Yuan Sheng Hospital, Yuanlin city, Changhua County, Taiwan; f Department of Physical Medicine and Rehabilitation, Yumin Medical Corporation Yumin Hospital, Caotun Town, Nantou County, Taiwan; g Branch of Physical therapy, Department of Physical Medicine and Rehabilitation, Changhua Christian Hospital, Changhua, Taiwan.

**Keywords:** lymphedema, manual lymphatic drainage, oral cancer, pain, range of motion, rehabilitation, sonographic measurement

## Abstract

There are clinical and statistical inconsistencies regarding early intervention with manual lymphatic drainage (MLD). The purpose of this study was to compare the short-term effect of early interventions with rehabilitation exercise versus MLD and rehabilitation exercise in terms of pain, range of motion (ROM) and lymphedema in patients with oral cancer after surgery.

**Methods:**

A total of 39 patients who underwent surgery from December 2014 to December 2018 participated in this randomized single-blind study. There were 20 patients in the rehabilitation (R) group and 19 in the MLD (M) plus rehabilitation group. The R group received 30 minutes of rehabilitation intervention; and the M group received 30 minutes of MLD, in addition to 30 minutes of rehabilitation intervention in a work day. Clinical measures, including the visual analog pain scale (VAS), ROM of the neck and shoulder, ultrasonography and face distance for lymphedema, and the Földi and Miller lymphedema scales, were assessed before surgery, before intervention and when discharged from the hospital.

**Results:**

The VAS pain score, ROM of the neck, and internal and external rotation of the right shoulder were significantly improved after the interventions. Right-face distance (*P = *.005), and skin-to-bone distance (SBD) of the bilateral horizontal mandible and left ascending mandibular ramus were significantly improved after the interventions. Left lateral flexion of the neck (*P = *.038) and SBD of the right ascending mandibular ramus (*P < *.001) in the MLD group showed more improvement than that of the rehabilitation group.

**Conclusion:**

Early intervention with MLD and the rehabilitation program were effective in improving ROM of the neck and controlling lymphedema in acute-phase rehabilitation. The preliminary findings suggest a potential therapeutic role for early intervention with MLD, in addition to rehabilitation exercise, in that they yielded more benefits in lymphedema control and improvement of ROM of the neck in acute care.

## 1. Introduction

Lip and oral cavity cancer is the 15^th^ most common cancer in the world,^[1,2]^ and oral cavity cancer is the most common cancer among middle-aged Taiwanese men.^[3]^ This age group is the most productive, so it would be of benefit to our nation’s productivity if the morbidity after head and neck cancer (HNC) could be reduced. Approximately 66% of HNC cases are diagnosed at advanced stages (III or IV).^[4,5]^ Speksnijder CM et al reported that neck dissection is related to deterioration of shoulder function, especially active shoulder abduction.^[6]^ Van der Molen L et al found that preventive rehabilitation in HNC patients, despite an advanced stage and burdensome treatment, seems helpful in reducing the extent and/or severity of the functional effects of concomitant chemoradiotherapy (CCRT).^[2]^ Do JH et al concluded that physical exercise has a positive effect on quality of life (QOL), including treating depression, fatigue, and anxiety in patients with HNC, and hospital-based exercise had a better effect than home-based exercise on the physical functioning of the neck and shoulder and on reducing pain.^[7]^

Effective curative treatment for HNC is surgery, radiation therapy (RT) and/or chemotherapy, in different combinations.^[8]^ Surgery and RT can disrupt the lymphatic structure and its functions, leaving HNC patients at high risk for developing secondary lymphedema.^[9,10]^ Existing evidence indicates that 12% to 54% of patients with HNC have developed secondary lymphedema.^[11]^ Chronic lymphedema may result in long-term cosmetic, functional, and psychosocial consequences.^[8,10,12]^ The current standard care for lymphedema is complex decongestive physiotherapy (CDP), which includes manual lymphatic drainage (MLD), compression therapy, exercise, and skin care.^[13]^ CDP can effectively reduce the lymphedema volume^[14,15]^ and improve QOL for breast cancer survivors.^[16]^ CDP is also used to treat head and neck lymphedema (HNL). Smith BG reported that 60% (439/733) of patients with HNL showed improvement after CDP, and he also suggested that HNL is distinct from the lymphedema that affects other sites, requiring adaptations in traditional methods of management and measurement.^[17]^ But evidence for the efficacy of all types of lymphedema therapy for HNL is limited by the paucity of large randomized controlled trials.^[18]^ Torres Lacomba M et al found that early physiotherapy (including MLD, massaging of scar tissue, and progressive active and action-assisted shoulder exercises) prevented lymphedema in women 1 year post-surgery for breast cancer.^[19]^ To the best of our knowledge, studies on the use of MLD to prevent morbidity in HNC patients are limited. Krisciunas GP et al used MLD (once weekly) with 5 patients during RT. The patients reported benefits with the use of MLD and that it lessened their throat pain during treatment sessions.^[20]^

There are still clinical and statistical inconsistencies among various studies on early intervention with MLD, so, in this study, we aimed to compare the short-term effect of early interventions with rehabilitation exercise versus MLD and rehabilitation exercise in terms of pain, range of motion (ROM) and lymphedema in patients with oral cavity cancer after surgery.

## 2. Materials and Methods

### 2.1. Study design and sample

This study used a randomized, single-blind design, and was conducted from December 2014 to December 2018. The study was approved, and ethical clearance was obtained from the hospital’s Institutional Review Board (IRB 131232). All participants provided written informed consent for their participation.

Patients with squamous cell carcinoma of the oral cavity who planned to undergo surgery at the Department of Oral and Maxillofacial Surgery, Changhua Christian Hospital, from December 2014 to December 2018, were invited to participate in this randomized controlled study. A total of 39 patients with oral cavity cancer were enrolled. Patients with a recurrence of cancer, an injured shoulder or neck, or with ROM of the shoulder less than 120 degrees or ROM of the neck less than 50 degrees were excluded. Patients were randomized into a rehabilitation group (R group) or a MLD plus rehabilitation group (M group) using computer-generated randomization. The R group received 30 minutes of rehabilitation intervention, including coughing, breathing exercises, and ROM of the neck and shoulder; the M group received 30 minutes of MLD, which may be directed at either anterior or posterior lymphatic drainage to axillary and neck lymph nodes, depending on the site of resection and subsequent scarring, in addition to 30 minutes of rehabilitation intervention in a work day. The rehabilitation intervention was performed by a physical therapist, and MLD was performed by another physical therapist (HY Huang). The intervention was carried out 7-10 days after surgery, and until the medical condition was stable, because most patients underwent extensive surgery and flap reconstruction. The intervention ended when the patient was discharged.

### 2.2. Measures

Demographic data including gender, age, body mass index (BMI), location and staging of cancer, surgical method, and number of removed lymph nodes were recorded. Participants underwent evaluation before surgery, before intervention and when discharged from the hospital. Measurements, except ultrasonography (US), were performed by a physical therapist, and the US measurement was performed by Dr Chen KL; both were blinded to the group allocation.

Clinical measures, including the visual analog pain scale (VAS), ROM of the neck (forward flexion, extension, rotation and lateral flexion) and shoulder (flexion, extension, abduction, internal rotation and external rotation), US and face distance for lymphedema, and the Földi and Miller lymphedema scales, were assessed. The Földi scale^[21]^ divided lymphedema into stages 0, 1, 2, and 3, and the Miller scale^[22]^ graded lymphedema from 0 to 4 depending on severity; both scales were used to assess the clinical stage of lymphedema. The skin-to-bone distance (SBD) at 6 locations in the face and neck (zygomatic arch, ascending mandibular ramus, and horizontal mandible of both sides) was recorded by US.^[23]^ The US measurement was performed using a device (Siemens, Acuson Antares PE) with a 10 to 13MHz linear scanner. For tape measurements, 7 anatomic marks, including tragus, mental protuberance, mouth angle, mandibular angle, nasal wing, internal eye corner, and external eye corner were chosen as the reference points; a sum of the 7 distances for each side were calculated according to Piso et al^[24]^ in evaluating head-neck edema.

### 2.3. Statistical analysis

Continuous variables in the demographic factors of the 2 groups were compared using *t* test statistics. Proportional distribution of demographic and clinicopathologic factors was compared using the 2-tailed chi-square test. The chi-square test of independence using Fisher’s exact test determined whether there was an association between the 2 groups. Data are presented as mean ± standard deviation (SD). The differences in the VAS pain score, ROM, lymphedema scale, face distance and US measurement of each evaluation were analyzed using generalized estimating equations (GEE). All analyses were performed using SPSS software version 22.0 for Windows (SPSS Inc., Chicago, IL). *P* values of < .05 were considered statistically significant.

## 3. Results

The 39 patients were randomized into 2 groups: 20 patients were placed in the R group and 19 in the M group. The mean age was 55.5 ± 11 years, the mean BMI was 24.5 ± 3.2 (kg/m^2^), and the number of intervention sessions was 9.2 ± 9.7. No patients received induction chemotherapy. There were no differences in age, BMI, tumor location, number of removed lymph nodes, methods of surgery, number of patients that received flap reconstruction, type of flap reconstruction, number of patients with a preserved spinal accessory nerve (SAN), internal jugular vein or sternomastoid muscle, stage of cancer, and intervention sessions in the 2 groups (Table 1). Thirty-four patients underwent flap reconstruction: 20 (13 in the R group, 7 in the M group) received radial forearm free flap reconstruction, and the other patients received an anterior lateral thigh flap, vastus lateralis myocutaneous flap or pectoralis major myocutaneous flap.

**Table 1:** Demographic and clinical characteristics of the study participants.

Variables	All cases in study	Rehabilitation group (SD)	MLD group (SD)	*P*
No	39	20	19	
Age (yr)	55.5 (11)	52.2 (9.8)	59 (11.3)	.99
BMI (kg/m^2^)	24.5 (3.2)	24.2 (3.1)	24.9 (3.3)	.75
Tumor location				.181
Right		10	4	
Left		4	9	
Bilateral		1	0	
Tongue		4	5	
Palate		1	1	
Stage				
I	10	7	3	.212
II	7	2	5	
III	5	4	1	
IVa	16	7	9	
IVb	1		1	
Surgery method RND	2	0	2	.54
mRND	8	4	4	
FND	8	3	5	
SND	13	8	5	
Bilateral ND	8	5	3	
SAN preserved	37	19	18	1
IJV preserved	32	18	14	.235
SCM preserved	24	12	12	1
Flap	34	18	16	.66
Flap type				.44
RFFF	20	13	7	
ALTF	6	3	3	
FOFF	1	0	1	
VLMC	5	2	3	
PMMC	2	0	2	
Lymph nodes removed	41 (19.2)	45.3 (22.8)	36.1 (13.5)	.06
Therapy sessions	9.2 (9.7)	7.8 (8)	10.7 (11.3)	.4

BMI = body mass index, MLD = manual lymphatic drainage, SAN = spinal accessory nerve.

The ROM of neck forward flexion, extension, rotation, and lateral flexion of both groups were deteriorated after surgery, but showed significant improvement after the interventions (Table 2). Left lateral flexion of the neck in the M group showed more improvement than that of the R group (*P* = .038) (Table 2). With regard to ROM of the shoulder, only impaired internal and external rotation of the right shoulder (*P* = .008, *P* = .005) was significantly improved after intervention (Table 2).

**Table 2:** Summary of measures of range of motion.

	Rehabilitation group (SD)			MLD group (SD)			Group *Time	Group	Time
Variables	Baseline	Before intervention	After intervention	Baseline	Before intervention	After intervention			
Neck Forward flexion	32.4(5.9)	11.9(9.1) ^‡^	23.1 (9.6) ^‡^	35.5 (9.2)	10.5 (8.7) ^‡^	28 (6.8) ^‡^	*P* = .42	*P* = .214	*P < *.001^‡^
Extension	33.3(10)	10.2(7.8) ^‡^	22.5(8.5) ^‡^	34.1 (8.5)	9.8 (8.2) ^‡^	20 (11) ^‡^	*P* = .806	*P* = .782	*P < *.001^‡^
Rotation									
Rt	45.4 (14.7)	10.6 (8.3) ^‡^	32.5 (14.1) ^‡^	48.1 (17.7) ^‡^	10 (7.8) ^‡^	30.8 (11.6)^‡^	*P* = .729	*P* = .463	*P < *.001^‡^
Lt	53.3 (19.3)	13.2 (11.3) ^‡^	37.5 (15.6) ^‡^	52.5 (21.1)	12.6 (10.8) ^‡^	35.8 (16.6) ^‡^	*P* = .511	*P* = .748	*P < *.001^‡^
Lat flexion									
Rt	30.6 (9)	9.8 (6.9) ^‡^	23.1 (4.6) ^‡^	32.7 (9.7)	10.4 (9.3) ^‡^	20 (4.5) ^‡^	*P* = .443	*P* = .93	*P < *.001^‡^
Lt	27.9 (9.2)	9.6 (7.2) ^‡^	20.4 (5.7) ^‡#^	34 (9.8)	8.7 (8.3) ^‡^	23.2 (6.2) ^‡#^	*P* = .094	*P* = .025^#^	*P < *.001^‡^
Rt shoulder									
Flexion	170.8(6.3)	157(9.4)	150(21.5)	167.3(8.6)	155.8 (12.2)	160.8 (11.3)	*P* = .105	*P* = .444	*P < *.001
Extension	73.8 (35)	52.6 (32)	58.8 (12.5)	58.1(11.5)	42.2 (18.7)	49.2(13.2)	*P* = .598	*P* = .33	*P = *.001
Abduction	170.4 (6.9)	147.5 (37.9)	153.1 (28.1)	168.5(12)	144.4 (25.8)	157.5 (10.4)	*P* = .527	*P* = .74	*P < *.001
Adduction	122.1 (19.8)	105.5 (8.6)	112.5 (7.6)	112 (18.2)	103.1 (15.3)	110 (14.8)	*P* = .587	*P* = .154	*P < *.001
Internal rotation	84.6 (9.2)	54.5 (24.8) ^‡^	73.8 (18.1) ^‡^	71.5 (11.8)	49.4 (26.6) ^‡^	64.2 (19.3) ^‡^	*P* = .492	*P* = .259	*P < *.001^‡^
External rotation	79.2 (7.6)	54.9 (20.4) ^‡^	73.1 (16.2) ^‡^	75.4(10.3)	52.4(22.6) ^‡^	67.5(12.1) ^‡^	*P* = .9812	*P* = .67	*P < *.001^‡^
Lt shoulder									
Flexion	170.4 (6.9)	158.5 (13.8) ^$^	150 (23.3)	164.2(11)	133.9(44.9) ^$^	142.5(33)	*P* = .142	*P* = .025^$^	*P < *.001
Extension	70.8 (29.6)	54.8 (26.9)	60.6 (8.2)	59.6 (10.7)	40.8 (22.2)	58.3 (19.4)	*P* = .365	*P* = .996	*P = *.05
Abduction	174.6 (4)	148.3 (33.5)	152.5 (35.3)	168.9(11.5)	138.1 (30.3)	140 (29.2)	*P* = .639	*P* = .735	*P < *.001
Adduction	115.8(6)	107.3(15)	113.8(11.9)	114.2(21.6)	103.9 (19.1)	115(15.5)	*P* = .837	*P* = .514	*P* = .003
Internal rotation	76.7(15.3)	52.5(27.4)	79.4(9.3)	75.8 (10)	51.7 (25.5)	70 (8.9)	*P* = .468	*P* = .638	*P* = .000
External rotation	81.3(5.7)	57.5(22.5)	78.1(5.3)	74.6 (12)	51.4 (20.4)	65 (16.1)	*P* = .962	*P* = .077	*P < *.001

MLD = manual lymphatic drainage.

**Figure 1.**
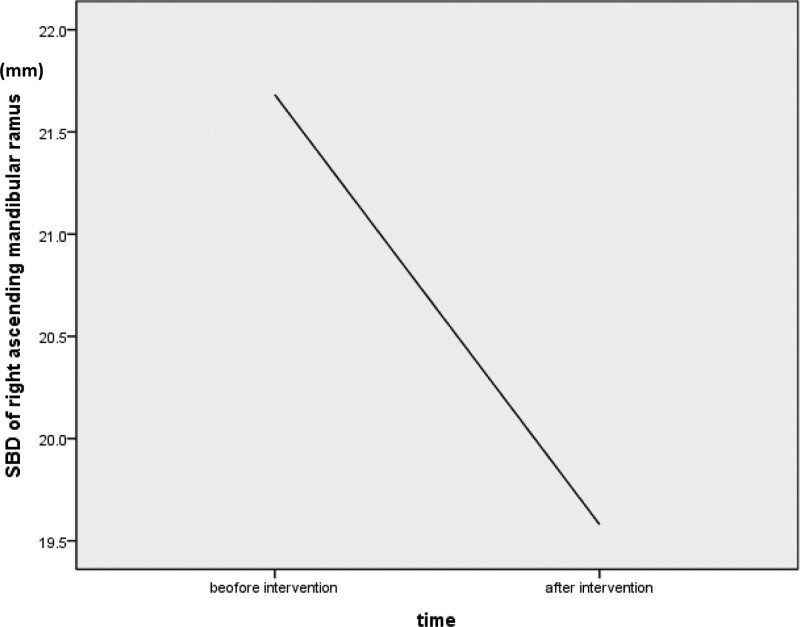
In MLD group, the lymphedema over right mandibular ramus was significantly improved after intervention. SBD = skin to bone distance, MLD = manual lymphatic drainage.

The VAS pain score was significantly improved in both groups after the interventions, with no difference between the 2 groups (Table 3). In the lymphedema evaluation, right facial distance showed improvement (*P* = .005), but there was no difference in the Foldi scale, the Miller score and left facial distance after therapy (Table 3). The SBD of the bilateral horizontal mandible and left ascending mandibular ramus was significantly improved after interventions in both groups (Table 3). The SBD of the right ascending and right horizontal mandibular ramus in the R group was poorer than that of the M group; this might be because the tumors of more patients in the R group were located in the right oral cavity. The SBD of the right ascending mandibular ramus in the M group was significantly improved after the interventions (*P < *.001) (Fig. 1), but there was no difference in the R group.

**Table 3:** Summary of measures of lymphedema and pain.

	Rehabilitation group			MLD group					
	Baseline	Before intervention	After intervention	Baseline	Before intervention	After intervention	Group *Time	Group	Time
VAS	0	3.9(2.6)	0.9(1.2)	0.5(1.2)	4.6(2.4)	0.5(1.2)	*P* = .786	*P* = .498	*P < *.001‡
Foeldi scale	0	1.1 (0.2)	0.9 (0.4)	0.3 (0.5)	1	1	*P* = .144	*P* = .172	*P < *.001
Miller grade	0	1.3 (0.6)	1 (0.5)	0.3 (0.5)	1.1 (0.2)	1	*P* = .123	*P* = .987	*P < *.001
Facial distances (cm)									
Rt	82 (2.9)	87.3 (10.5)	86.9 (6.8)	80 (6.2)	83.4 (11.1)	80.6 (9.1)	*P* = .462	*P* = .224	*P < *.001‡
Lt	82.5 (3.8)	86.4 (5)	85.5 (5.8)	81.4 (6.1)	85 (10.7)	87.1 (8.2)	*P* = .29	*P* = .258	*P < *.001
SBD (mm) Rt zygomatic arch		9.2 (1.9)	10.3 (3.6)		8.3 (1.1)	8.6 (1.7)	*P* = .387	*P* = .083	*P* = .133
Rt ascending mandibular ramus		30.3(10.4) $	23.1(4.9) #		21.7 (4.5)$‡	19.6 (5)#‡	*P* = .659	*P < *.0001$#	*P* = .106
Rt horizontal mandible		29.5(6.6) $‡	22.4(5.5) #‡		20.9 (4.8) $‡	18.8 (5.5) #‡	*P* = .02$#	*P < *.0001$#	*P < *.0001‡
Lt zygomatic arch		8.6(1.2)	9.7 (2.5)		9.6 (6.1)	9.8 (2.6)	*P* = .639	*P* = .602	*P* = .414
Lt ascending mandibular ramus		25.7(6.5) ‡	20.7 (1.1) ‡		24 (8.6) ^‡^	22.3 (4.6) ‡	*P* = .888	*P* = .322	*P < *.001‡
Lt horizontal mandible		24.9(7) ‡	18.2(3.6) ‡		23.5 (8.6) ‡	24.5 (4.8) ‡	*P* = .574	*P* = .68	*P < *.001‡

MLD = manual lymphatic drainage, SBD = skin-to-bone distance, VAS = visual analog pain scale.

In the Generalized Linear Models analysis:

‡ *P < *.05 the data before intervention compared with after intervention.

$ *P < *.05 the data of the rehabilitation group compared with that of the MLD group before intervention.

# *P < *.05 the data of the rehabilitation group compared with that of the MLD group after intervention.

*P* < .05 the data of the rehabilitation group compared with that of the MLD group.

## 4. Discussion

In this study, we showed that early intervention with MLD in addition to rehabilitation exercise might have more benefit than rehabilitation exercise alone in treating lymphedema and ROM of the neck in acute-phase rehabilitation, although the effect of a longer intervention in the M group (60 min vs 30 min) should be considered.

After 9 intervention sessions, the pain, ROM of the neck, and internal and external rotation of the right shoulder were significantly improved. Bauml J et al reported that an appropriate exercise program would be of benefit to patients with HNC.^[25]^ Do JH et al found that hospital-based exercise had a better effect than home-based exercise on physical functioning of the neck and shoulder and on reducing pain.^[7]^ But, Su TL reported that a home-based program for patients with HNC was not inferior to an outpatient program for improving QOL, functional capacity, and shoulder ROM.^[26]^ The difference in outcome between these 2 studies is due to the dissimilar participants: those in the Do JH study were more specific subjects with shoulder dysfunction after SAN injury, and those in the Su TL study were usual HNC patients 6 months after therapy. When treating HNC patients with a specific dysfunction or patients in the acute phase after surgery, we could conclude that a hospital-based rehabilitation program is more efficient.

Although there was improvement after intervention, most participants still had moderately limited ROM of the neck. Deng J et al^[27]^ reported that most participants had mildly to moderately decreased neck ROM in 6 directions 3 months or more after HNC treatment. This means that 7-10 interventions are not enough to recover impaired neck ROM. The SAN of most patients was preserved, but for the most part, ROM of bilateral shoulders did not show improvement when the patients were discharged from the hospital. Chepeha DB reported that patients undergoing modified radial neck dissection had significantly worse shoulder function than patients with selective neck dissection.^[28]^ In a 1-year prospective cohort study of 145 oral cancer patients, Speksnijder CM found that more extended neck dissections induced greater deterioration in neck function and significantly lower maximal abduction of the shoulder.^[6]^ In shoulder abduction, trapezius muscle activity is needed to rotate upward and stabilize the scapula. Extensive neck dissection will disturb the functioning of the SAN and lead to a loss of function of the trapezius muscle, even with preservation of the SAN.^[6,29,30]^ According to the above finding, shoulder dysfunction would require more time for recovery than ROM of the neck, as in our results. So, the inpatient rehabilitation program should extend to regular outpatient rehabilitation until there is full recovery of shoulder and neck functioning.

The improvement in left neck lateral flexion in the M group was better than that in the R group. This revealed MLD had an additional effect on ROM of the neck in the rehabilitation program. Doke KN reported that manual lymphatic decongestion and skilled fibrotic techniques are associated with objective improvements in ROM, neck circumference, and pain scores in HNC patients after at least 3 months of lymphedema therapy.^[31]^

The incidence of post-treatment lymphedema in HNC patients varied from 12% to 54%.^[11]^ There are currently no standard criteria or a grading system for lymphedema in HNC, although various tools have been used in previous studies.^[17,24,32–34]^ In this study, we chose to use Foldi’s scale, Miller’s score, and tape and US measurements to assess lymphedema. Only right-face distance and SBD on US showed a difference after the interventions. US measurement of SBD is more sensitive to detecting lymphedema change than the scale system or face distances. Lymphedema was significantly improved after 2 interventions, and MLD therapy showed more lymphedema reduction at the right ascending mandible ramus. This means MLD had a greater effect on reducing lymphedema than the traditional rehabilitation program. In this study, we actually found evidence of the effectiveness of MLD in improving acute HNL, but evidence validating the goal of MLD in preventing the long-term morbidity of HNC is limited. The improvement in lymphedema and ROM of the neck on the right side (SBD at the right ascending mandible ramus and left lateral flexion of the neck) in the M group might be due to the existence of more tumors in the left oral cavity and less disruption of the right oral cavity, leading to improvements on the right side of the body.

Pain is common in HNC patients, and 36% of patients still have pain at 6 months after treatment.^[35]^ The pain was significantly improved after the interventions, and the pain scale showed improvement from moderate pain (VAS 3–5) to below 1. But our participants received both the interventions and medicine control, and together, they were effective in relieving HNC pain after surgery. In the future, a control group is needed to reveal the actual goal of MLD and rehabilitation exercise in treating acute post-surgery pain. MLD did not have an effect on pain control beyond the rehabilitation program. Uher EM et al also reported that manual lymph drainage provides no additional benefit when applied in conjunction with an intensive exercise program to reduce complex regional pain syndrome type I.^[36]^

The limitations in our study are the small number of participants, the intervention time spent with each group was not equal, and there was no control group to consider potential spontaneous recovery (however, it is unethical to set up a control group when rehabilitation interventions are regularly arranged). Further investigations that include more patients, the same intervention time, and longer follow-up periods to assess the effect of early interventions on preventing further morbidities should be carried out.

## 5. Conclusion

In conclusion, both MLD and the rehabilitation program were effective interventions to improve ROM and lymphedema in acute-phase rehabilitation. The preliminary findings, although limited, also suggest a potential therapeutic role for early intervention with MLD, in addition to rehabilitation exercise, in that they yielded more benefits in lymphedema control and improvement of ROM of the neck in acute care.

## Author contributions

**Conceptualization:** Kuo-Yang Tsai, Su-Fen Liao, Hao-Wei Tang.

**Data curation:** Kuo-Yang Tsai, Su-Fen Liao, Kuan-Lin Chen, Hsin-Ya Huang.

**Formal analysis:** Su-Fen Liao.

**Funding acquisition:** Su-Fen Liao.

**Investigation:** Kuo-Yang Tsai, Kuan-Lin Chen, Hao-Wei Tang, Hsin-Ya Huang.

**Methodology:** Kuo-Yang Tsai, Su-Fen Liao, Kuan-Lin Chen, Hao-Wei Tang, Hsin-Ya Huang.

**Project administration:** Kuo-Yang Tsai, Su-Fen Liao, Kuan-Lin Chen.

**Supervision:** Kuo-Yang Tsai, Su-Fen Liao.

**Validation:** Su-Fen Liao, Hao-Wei Tang, Hsin-Ya Huang.

**Visualization:** Su-Fen Liao, Hao-Wei Tang.

**Writing – original draft:** Su-Fen Liao.

**Writing – review & editing:** Kuo-Yang Tsai, Su-Fen Liao, Kuan-Lin Chen, Hao-Wei Tang.
